# Efficacy of [^18^F]PSMA-1007 PET/CT in Primary Staging of Prostate Carcinoma: A Systematic Review and Metaanalysis

**DOI:** 10.2967/jnumed.125.269818

**Published:** 2026-01

**Authors:** Kambiz Rahbar, Frederik L. Giesel, Ken Herrmann, Mijin Yun, Tadashi Watabe, Ines Rudolph, Alexander Hoepping, Tobias Maurer

**Affiliations:** 1 Department of Nuclear Medicine, University Hospital Muenster, Muenster, Germany;; 2 West German Cancer Center, Muenster and Essen, Germany;; 3 Department of Nuclear Medicine, University Hospital Duesseldorf, Duesseldorf, Germany;; 4 Department of Radiology, Graduate School of Medicine, Osaka University, Osaka, Japan;; 5 Department of Nuclear Medicine, University Hospital Essen, Essen, Germany;; 6 Department of Nuclear Medicine, Yonsei University, Seoul, South Korea;; 7 ABX Advanced Biochemical Compounds, Biomedizinische Forschungsreagenzien GmbH, Radeberg, Germany; and; 8 Department of Urology and Martini-Klinik Prostate Cancer Center, University of Hamburg-Eppendorf, Hamburg, Germany

**Keywords:** prostate carcinoma, PET/CT, [^18^F]PSMA-1007, Radelumin, staging

## Abstract

Staging of prostate carcinoma (PCa) still largely relies on histopathologic examination of prostate tissue. In the last few years, PET/CT with radiotracers that target the prostate-specific membrane antigen (PSMA) has emerged as a noninvasive and sensitive method for staging of PCa. Compared with [^68^Ga]PSMA-11, [^18^F]PSMA-1007 is a relatively new radiotracer for PSMA PET/CT with favorable characteristics such as a longer physical half-life, reduced bladder background uptake, and improved availability due to production off-site. The objective of this systematic review and metaanalysis is to summarize the efficacy of [^18^F]PSMA-1007 in primary T, N, and M staging of PCa in comparison to histopathology. **Methods:** Clinical trials on primary staging of PCa with [^18^F]PSMA-1007 (both prospective and retrospective studies) were identified by a systematic search in PubMed. Relevant literature used histopathology as a comparator and reported discrete values for sensitivity and specificity. A metaanalysis assessed differences in diagnostic parameters. **Results:** Nineteen studies were included in this review: 10 studies reported on T staging (739 patients), 8 studies reported on N staging (865 patients), and 1 study reported on M staging (79 patients). For T staging, our metaanalyses of extraprostatic extension on a patient level based on 3 studies revealed a pooled sensitivity of 54% (95% CI, 46%–63%) and a pooled specificity of 92% (95% CI, 76%–98%). For N staging, our metaanalyses on detection of lymph node metastases on a patient level based on 5 studies revealed a pooled sensitivity of 42% (95% CI, 28%–57%) and a pooled specificity of 94% (95% CI, 90%–97%). In terms of sensitivity for M staging on a patient level, [^18^F]PSMA-1007 PET/CT outperformed all other tested conventional imaging modalities. **Conclusion:** PET/CT imaging with [^18^F]PSMA-1007 provides high sensitivity and specificity in T, N, and M staging of PCa when compared with histopathology. It offers the possibility to perform noninvasive primary T, N, and M staging before treatment in a single procedure.

## Visual Abstract

**Figure:**
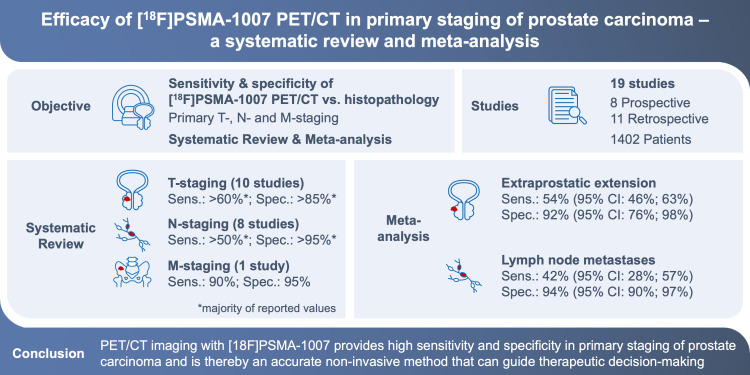

According to the 2023 cancer statistics for men in the United States, prostate carcinoma (PCa) is the most common type of cancer and the second leading cause of cancer-related death (1). Risk factors for PCa include family history, ethnicity, age, obesity, and other environmental factors, and PCa usually affects men over 45 y old (2). As localized PCa is usually asymptomatic, screening for PCa for men more than 45–50 y old is recommended by guidelines (3,4). Measurement of prostate-specific antigen levels in the blood is applied for early screening (3,4). When prostate-specific antigen levels are repeatedly elevated, further diagnostic examinations are performed, such as multiparametric MRI (mpMRI) and, to confirm diagnosis, prostate biopsy (3,4). To assess prognosis and tumor aggressiveness, PCa is graded by applying the Gleason score or the International Society of Urological Pathology grade group system on the histologic biopsy specimens (3–5).

PCa is best staged with the TNM system of the Union for International Cancer Control (3,4,6). In PCa, TNM staging is performed before treatment by clinical assessment and after treatment by pathologic assessment of histologic specimens from prostatectomy based on the PCa staging manual of the American Joint Committee on Cancer (7). Although the latter is the reference standard for definitive staging, therapeutic decision-making has to rely on clinical TNM staging; therefore, initial staging should be as accurate as possible. Although cT staging is to date still based on digital rectal examination findings only (3,8) imaging methods such as CT, mpMRI, and bone scintigraphy have been used routinely for decades for T staging as well as for N and M staging in PCa (3,4).

An alternative to those conventional imaging procedures is targeted imaging with PET, applied mostly in combination with CT but also in combination with MRI (9). PET imaging for PCa uses a radiotracer that specifically binds to the prostate-specific membrane antigen (PSMA) (9). PSMA is highly expressed on PCa tumor cells, in both primary and metastatic lesions, making it an ideal target for imaging (10,11). Several PSMA-directed radiotracers are already approved and used in clinical practice—for example, gallium-based radiotracers such as [^68^Ga]PSMA-11 and fluorine-based radiotracers such as [^18^F]DCFPyL or [^18^F]PSMA-1007 (9).

[^18^F]PSMA-1007 offers several advantages over gallium-based radiotracers. [^18^F]PSMA-1007 has a longer physical half-life than [^68^Ga]PSMA-11 (110 min vs. 68 min) and provides a PET image of improved resolution, which enables better detection of smaller lesions (12). In addition, [^18^F]PSMA-1007 offers advantages regarding availability and costs, as it can be produced off-site with high purity and in high yield (cyclotron production) and transported to the respective sites for use, whereas [^68^Ga]PSMA-11 has to be labeled on-site (generator production of ^68^Ga) (13). However, the most striking advantage of [^18^F]PSMA-1007 is that it is excreted via the hepatobiliary route, facilitating evaluation of the pelvic region (with low bladder background uptake), which might be obscured when using tracers with a renal excretion such as [^68^Ga]PSMA-11 or [^18^F]DCFPyL (12).

In this systematic review, we report on the efficacy of [^18^F]PSMA-1007 in primary staging of PCa (T, N, and M staging). We performed a systematic literature search and included studies that compared results from PET/CT or PET/MRI using [^18^F]PSMA-1007 with histopathologic analysis as a reference standard to assess the specificity and sensitivity of primary PCa staging with [^18^F]PSMA-1007.

## MATERIALS AND METHODS

### Systematic Literature Research

A systematic literature review was conducted on July 4, 2024. Relevant published studies were identified through a PubMed/MEDLINE search. The following combination of keywords was used: (prostate cancer[Title/Abstract] OR prostate carcinoma[Title/Abstract]) AND (PSMA-1007[Title/Abstract] OR radelumin[Title/Abstract] OR F-PSMA[Title/Abstract]) AND (clinical[Title/Abstract] OR study[Title/Abstract] OR trial[Title/Abstract] OR patient*[Title/Abstract]). The identified studies were initially screened for eligibility on the basis of the title and abstract by one author. The full texts of the potentially relevant publications were retrieved and first checked by one author. The results regarding inclusion and exclusion were independently checked by a second author, and discrepancies were resolved by consensus. All included clinical studies analyzed the efficacy of [^18^F]PSMA-1007 PET/CT or PET/MRI with histopathologic analysis as the reference standard and reported on sensitivity and specificity. Exclusion criteria were as follows: not a clinical study (e.g., case reports, reviews), no staging or no histopathology as reference standard, language other than English, study not conducted in the primary staging setting but in the recurrent setting (relapse/biochemical recurrence), use of a tracer different from [^18^F]PSMA-1007, and no reporting on sensitivity and specificity with reference to histopathology. One author extracted the following data from each included study: study design, number of patients or participants, information on tumor location (T staging: intraprostatic, laterality, extraprostatic extension [EPE], seminal vesicle invasion [SVI]; N staging: lesion-based and patient-based N staging; M staging), information on tumor grading, and sensitivity and specificity with reference to histopathologic analysis. The extracted data were then checked by a second author. This review was not prospectively registered.

### Metaanalysis

To further analyze the sensitivity and specificity of [^18^F]PSMA-1007 PET/CT in staging of PCa, we performed metaanalyses on N staging and T staging. N staging was reported on a patient level or on a lesion level. Separate metaanalyses were conducted for the 2 approaches. Depending on the publication, T staging was reported by segment, for EPE or SVI. Separate metaanalyses were performed for the different approaches and thresholds. Within the metaanalyses, the DerSimonian–Laird estimate was used to estimate between-study variance. Studies were pooled on the basis of the inverse variance method.

The true positives, false negatives, false positives, and true negatives were extracted from the publications. Then, the parameter estimates were calculated along with Clopper–Pearson exact 95% CIs. Metaanalyses for sensitivity and specificity were computed using the R-package “meta” (14). In metaanalyses containing 2 studies, fixed-effects estimates were computed, whereas in metaanalyses with 3 or more studies, random-effects estimates were generated. For metaanalyses in which the assumption of homogeneity was violated (*P* ≤ 0.05), sensitivity analyses were performed by excluding the most extreme study whenever possible.

## RESULTS

### Results of the Systematic Literature Search

**FIGURE 1.**
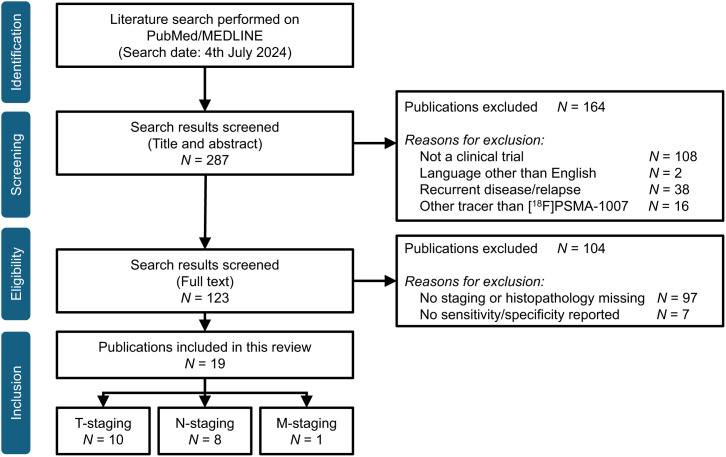
Results of literature search and exclusion/inclusion of studies for this review.

Our search identified 287 articles, of which 268 were excluded, 164 through title/abstract screening and 104 through full-text screening. In the title/abstract screening, 108 studies were excluded because they were not clinical trials, 2 because the language of the publication was not English, 38 because they were performed in the recurrent setting (relapse/biochemical recurrence), and 16 because they used a tracer other than [^18^F]PSMA-1007. In the full-text screening, 97 studies were excluded because no staging was performed or histopathology as a reference was missing, and 7 studies were excluded because they did not report on sensitivity and specificity with reference to histopathology. In total, 19 studies with 1,420 patients were included in this systematic review (Fig. 1).

### [^18^F]PSMA-1007 PET/CT in T Staging of PCa

Ten of the included studies, with a total of 739 patients, analyzed the sensitivity and specificity of [^18^F]PSMA-1007 PET/CT in T staging of PCa with reference to histopathology. Thirteen of the 20 reported sensitivities were more than 60% (range, 23%–100%). Thirteen of the 20 reported specificities were more than 85% (range, 10%–100% [Supplemental Table 1; supplemental materials are available at http://jnm.snmjournals.org]). In the following paragraphs, the main results of the 10 studies are sorted by [^18^F]PSMA-1007 PET/CT with reference to histopathology (2 studies), by [^18^F]PSMA-1007 PET/CT versus [^68^Ga]PSMA-11 with reference to histopathology (2 studies), and by [^18^F]PSMA-1007 PET/CT versus mpMRI with reference to histopathology (6 studies) in T staging of PCa.

Two retrospective studies, from Hong et al. (15) and Luo et al. (16), with 218 patients in total evaluated [^18^F]PSMA-1007 PET/CT solely with histopathology and found high sensitivities of approximately 91%, whereas the specificities—at 65.3% and 50%, respectively—were lower. In the study of Luo et al., specificity could be increased to 95.8% by adjusting the cutoff SUV_max_ to 8.3, with a corresponding sensitivity of 74.4%.

Two studies, by Kuten et al. (17) and Hoffmann et al. (18), compared the performance of 2 PET tracers, that is, [^18^F]PSMA-1007 and [^68^Ga]PSMA-11, with histopathology as a reference. In both studies, [^18^F]PSMA-1007 demonstrated markedly higher sensitivity than [^68^Ga]PSMA-11 (100% and 62% vs. 85.7% and 54%) and similar specificity to [^68^Ga]PSMA-11 (90.9% and 85% vs. 98.2% and 91%).

Six studies compared [^18^F]PSMA-1007 PET/CT with mpMRI, with histopathology as a reference. Two smaller studies, by Kesch et al. (19) and Zamboglou et al. (20) (both on 10 patients), reported the performance of [^18^F]PSMA-1007 PET/CT and mpMRI in staging of prostatic lesions. The study by Kesch et al. found that when considering total agreement with histopathology, sensitivity was higher for mpMRI (86% vs. 71%), whereas specificity was higher for [^18^F]PSMA-1007 PET/CT (81% vs. 64%). The study of Zamboglou et al. also evaluated the effect of 2 different coregistration pathways for image analysis. For both coregistration pathways, sensitivity was higher for [^18^F]PSMA-1007 PET/CT than for mpMRI (83%–84.5% vs. 60%–69%), whereas specificity was similar (74%–93.8% vs. 100%).

Two studies, by Privé et al. (21) and Tang et al. (22), evaluated the performance of [^18^F]PSMA-1007 PET/CT and mpMRI in staging EPE and SVI. In the study of Privé et al., [^18^F]PSMA-1007 PET/CT showed higher or equal specificity for EPE and SVI (83% and 85% vs. 67% and 85%), whereas sensitivity was lower with [^18^F]PSMA-1007 PET/CT in the case of EPE (41% vs. 80%) and higher in the case of SVI (80% vs. 50%) when compared with mpMRI. Interestingly, the study by Tang et al. showed similar trends, with [^18^F]PSMA-1007 PET/CT exhibiting higher specificity for both EPE and SVI (100% and 100% vs. 89.3% and 98.1%), lower sensitivity for EPE (23% vs. 44.6%), and higher sensitivity for SVI (51.9% vs. 40.7%).

Two larger prospective studies (214 patients in total), by Exterkate et al. (23) and Mookerji et al. (24), reported the performance of [^18^F]PSMA-1007 PET/CT and mpMRI in staging of prostatic lesions as well as EPE and SVI. In the study of Exterkate et al., sensitivities and specificities for [^18^F]PSMA-1007 PET/CT were numerically lower than those for mpMRI for EPE (patient-based EPE: sensitivity of 53% vs. 58%, specificity of 68% vs. 80%; lesion-based EPE: sensitivity of 45% vs. 55%, specificity of 85% vs. 90%). Regarding SVI, sensitivities for [^18^F]PSMA-1007 PET/CT were numerically higher than those for mpMRI, whereas specificities were similar (patient-based SVI: sensitivity of 54% vs. 31%, specificity of 90% vs. 93%; lesion-based SVI: sensitivity of 47% vs. 33%, specificity of 94% vs. 96%). In the study of Mookerji et al., [^18^F]PSMA-1007 PET/CT outperformed mpMRI in terms of sensitivity for PCa laterality, EPE, and SVI (69%, 58%, and 57% vs. 39%, 33% and 33%, respectively). Specificity for EPE and SVI was similar between the 2 methods (90% and 90% vs. 96% and 97%, respectively), whereas specificity for PCa laterality was markedly lower with [^18^F]PSMA-1007 PET/CT (27% vs. 93%).

### Metaanalyses of [^18^F]PSMA-1007 PET/CT in T Staging of PCa

To further analyze the sensitivity and specificity of [^18^F]PSMA-1007 PET/CT in T staging of PCa, we performed metaanalyses for T staging separately for EPE (patient level) and SVI (patient level) as well as for T staging on a lesion level. Four publications reported T staging for EPE on a patient level. A metaanalysis of sensitivities showed heterogeneity (*P* = 0.0002) (Supplemental Fig. 1). The exclusion of the publication of Tang et al. in a sensitivity analysis led to homogeneity (*P* = 0.44). The metaanalytic sensitivity for T staging of EPE on a patient level was 0.54 (95% CI, 0.46–0.63) (Supplemental Fig. 2). The metaanalysis of the reported specificities of the 4 studies for T staging of EPE also displayed substantial heterogeneity (*P* = 0.0075) (Supplemental Fig. 3). Because of the exclusion of the publication by Exterkate et al., homogeneity was reached (*P* = 0.18). The metaanalytic specificity in T staging of EPE on a patient level was 0.92 (95% CI, 0.76–0.98) (Supplemental Fig. 4). Four studies reported T staging for SVI on a patient level. The studies were found to be homogeneous (*P* = 0.51). The metaanalytic sensitivity in T staging of SVI on a patient level was 0.57 (95% CI, 0.45–0.68) (Supplemental Fig. 5). In terms of specificity, the 4 studies reporting on T staging for SVI on a patient level showed heterogeneous results (*P* = 0.03). The exclusion of publications from the metaanalysis did not lead to homogeneous results (Supplemental Figs. 6–8). Metaanalyses of sensitivity and specificity in T staging on a lesion level did not reach homogeneity (Supplemental Figs. 9–12).

### [^18^F]PSMA-1007 PET/CT in N Staging of PCa

Eight of the included studies analyzed the sensitivity and specificity of [^18^F]PSMA-1007 PET/CT in N staging of PCa in a total of 865 patients: some analyzed sensitivity and specificity on a patient level, some on a lesion level, and some on both (Supplemental Table 2). In the following paragraphs, the main results of the 8 studies are sorted by [^18^F]PSMA-1007 PET/CT with histopathology as a reference standard (4 studies), by [^18^F]PSMA-1007 PET/CT versus [^68^Ga]PSMA-HBED.CC with histopathology as a reference standard (1 study), and by [^18^F]PSMA-1007 PET/CT versus mpMRI or whole-body MRI (WBMRI) with diffusion-weighted imaging (DWI) and CT with histopathology as a reference standard (3 studies) in N staging of PCa.

Four studies, by Giesel et al. (25), Sprute et al. (26), Hermsen et al. (27), and Ingvar et al. (28), evaluated the sensitivity and specificity of [^18^F]PSMA-1007 PET/CT compared solely with histopathology. The small pilot study by Giesel et al. reported high sensitivity (94.7%) and specificity (100%) for the detection of pelvic lymph node (LN) metastases. In the study of Sprute et al., specificities both on a patient level and on a lesion level were also high (>99%), whereas sensitivities were slightly lower (patient level: 73.5%; lesion level: 71.2%). When lesions only 3 mm or larger were considered, sensitivity was higher and specificity was about the same (lesion-level sensitivity of 81.7% and specificity of 99.6%; patient-level sensitivity of 85.9% and specificity of 99.5%). In the study of Hermsen et al., [^18^F]PSMA-1007 PET/CT similarly showed high specificity (patient level, 89.9%; lesion level, 97.7%), although sensitivity was rather low (patient level, 53.3%; lesion level, 12.9%). The study of Ingvar et al. showed a similar picture, with high specificities (patient-level LNs of all sizes: 96.2%; lesion-level LNs ≥ 3 mm: 96.7%) but somewhat lower sensitivities (patient-level LNs of all sizes: 26.9%; lesion-level LNs ≥ 3 mm: 53.8%).

In a study by Baas et al. (29), PET/CT was performed with [^18^F]PSMA-1007 or with [^68^Ga]PSMA-HBED-CC as the tracer; most patients received [^18^F]PSMA-1007 (*n* = 175 vs. *n* = 38). Similar to Hermsen et al. and Ingvar et al., specificity for detection of pN1 disease was high (84%), whereas sensitivity was low (29%).

Tang et al. (22) and Mookerji et al. (24) compared [^18^F]PSMA-1007 PET/CT with mpMRI for N staging, Malaspina et al. (30) compared [^18^F]PSMA-1007 PET/CT with WBMRI (including DWI) and with standard CT. In all studies, [^18^F]PSMA-1007 PET/CT outperformed MRI in terms of sensitivity (62.5%, 50%, and 87% vs. 25%, 25%, and 37% respectively), whereas specificities were similar (90.4%, 98%, and 98% vs. 93%, 100%, and 98%, respectively).

### Metaanalyses of [^18^F]PSMA-1007 PET/CT in N Staging of PCa

**FIGURE 2.**
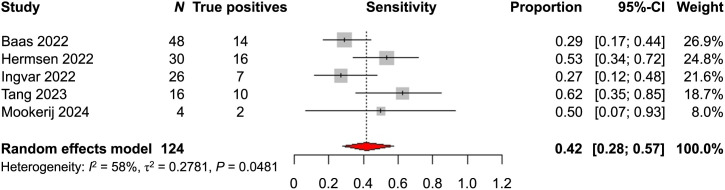
Metaanalysis of sensitivity of [^18^F]PSMA-1007 PET/CT in N staging on patient level, sensitivity analysis.

To further analyze the sensitivity and specificity of [^18^F]PSMA-1007 PET/CT in N staging of PCa, we performed metaanalyses for N staging separately on a patient level and on a lesion level. Six studies reported sensitivity and specificity for N staging on a patient level. The assumption of homogeneity was found to be violated in the metaanalysis on sensitivity (*P* < 0.0001) (Supplemental Fig. 13); thus, a sensitivity analysis was performed excluding the publication by Malaspina et al. The sensitivity analysis based on 5 studies displayed a near-homogeneous outcome (*P* = 0.0481), with no study lending itself to further exclusion. The metaanalytic sensitivity in N staging on a patient level was 0.42 (95% CI, 0.28–0.57) (Fig. 2).

**FIGURE 3.**
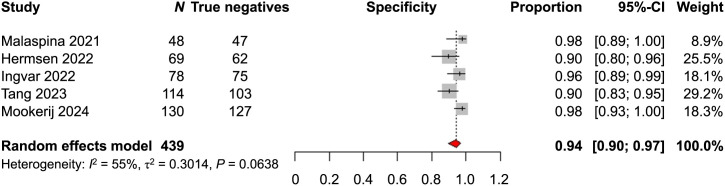
Metaanalysis of specificity of [^18^F]PSMA-1007 PET/CT in N staging on patient level, sensitivity analysis.

As for specificity, the outcomes of the 6 studies were also not found to be homogeneous (*P* = 0.0023) (Supplemental Fig. 14); thus, a sensitivity analysis was performed excluding the study of Baas et al. The metaanalytic specificity in N staging on a patient level was 0.94 (95% CI, 0.90–0.97) (Fig. 3).

Three studies reported N staging on lesion level. The resulting metaanalysis of reported sensitivities including all 3 studies showed heterogeneity (*P* < 0.0001) (Supplemental Fig. 15); thus, a sensitivity analysis was performed excluding the publication of Hermsen et al. The metaanalytic sensitivity in N staging on a lesion level was 0.73 (95% CI, 0.64–0.80) (Supplemental Fig. 16).

The metaanalysis of specificities on lesion level also showed heterogeneity (*P* = 0.0002), but because of close results, none of the studies lent itself to exclusion. The small *P* value is probably more a result of extremely high sample sizes than a sign of lack of homogeneity. The metaanalytic specificity in N staging on a lesion level was 0.99 (95% CI, 0.97–1.00) (Supplemental Fig. 17).

### [^18^F]PSMA-1007 PET/CT in M Staging of PCa

One study, by Anttinen et al. (31), evaluated the performance of [^18^F]PSMA-1007 PET/CT in M staging of PCa. This prospective study compared 5 imaging modalities for the detection of metastases in 79 patients with high-risk PCa, that is, ^99m^Tc-hydroxymethylene diphosphonate planar bone scintigraphy, CT, ^99m^Tc-hydroxymethylene diphosphonate SPECT/CT, 1.5-T WBMRI using DWI, and [^18^F]PSMA-1007 PET/CT. Sensitivities and specificities were determined by 2 readers in an optimistic and pessimistic analysis regarding the handling of equivocal findings. In terms of sensitivity for M staging on the patient level, [^18^F]PSMA-1007 PET/CT outperformed all other imaging modalities both in an optimistic analysis ([^18^F]PSMA-1007 PET/CT: 90%/95%; CT: 57%/43%; SPECT/CT: 67%/57%; WBMRI + DWI: 67%/52%) and in a pessimistic analysis ([^18^F]PSMA-1007 PET/CT: 86%/95%; CT: 43%/33%; SPECT/CT: 52%/33%; WBMRI + DWI: 67%/43%).

### PET/MRI with [^18^F]PSMA-1007 in Staging of PCa

The combination of PSMA PET with MRI has the possibility to overcome limitations of CT such as low resolution for soft tissues and can provide excellent diagnostic value for PCa imaging (32,33), although ubiquitous use of PSMA PET/MRI may still be limited because of availability issues (33). Several studies using the tracer [^68^Ga]PSMA-11 for PET/MRI have already been conducted (34–36).

We identified 2 studies that evaluated the performance of PET/MRI using [^18^F]PSMA-1007 in staging of PCa in recent years. Zeng et al. (12) included 29 patients with suspected PCa and compared the performance of [^18^F]PSMA-1007 PET/MRI with biparametric MRI in T staging of PCa. Sensitivities and specificities for detection of PCa were calculated on the basis of the histopathology of biopsy specimens after imaging. [^18^F]PSMA-1007 PET/MRI outperformed biparametric MRI in terms of sensitivity (94.74% vs. 92.11%) and specificity (100% vs. 50%), with an overall statistically significant better diagnostic performance (*P* = 0.0029). In a study by Ali et al. (32), 46 patients with pathologically verified intermediate- or high-risk PCa underwent [^18^F]PSMA-1007 PET/MRI with dedicated pelvic high-resolution mpMRI. T staging, N staging, and M staging were evaluated, with dedicated analysis of urethral bladder invasion, SVI, neurovascular bundle invasion, rectal invasion, LN metastases (local and distant), bone metastases, lung metastases, liver metastases, and adrenal metastases. [^18^F]PSMA-1007 PET/MRI showed excellent sensitivities and specificities, often higher than those for mpMRI or WBMRI (for distant metastases). PET/MRI demonstrated better agreement regarding TNM staging with histopathology than did mpMRI/WBMRI, with concordance with T, N, and M stages in 40, 41, and 36 patients, respectively.

### Safety of ^18^F-PSMA-1007

The safety of [^18^F]PSMA-1007 has been studied extensively. So far, there have been no reports of considerable adverse events related to the use of [^18^F]PSMA-1007. Within the multicenter randomized phase 3 ABX-301 trial comparing [^18^F]PSMA-1007 with ^18^F-fluorocholine for localization of PCa biochemical recurrence—a study that included 191 patients—there were no adverse events reported that were considered attributable to [^18^F]PSMA-1007 (37). The European summary of product characteristics of [^18^F]PSMA-1007 further states that there were no undesirable effects reported in the literature from use in more than 1,000 patients (38).

## DISCUSSION

This systematic review provides an overview of the efficacy of [^18^F]PSMA-1007 for PET-based staging of PCa based on sensitivity and specificity against the benchmark of histopathology. In general, PSMA-directed PET imaging has advantages over histopathology in PCa staging, as PSMA PET can deliver comprehensive information on the primary tumor, LNs, and distant metastases, whereas histopathology can deliver only limited local information. This makes PSMA PET a comprehensive all-in-one imaging modality for T, N, and M staging combined. Also, staging with PSMA PET has the advantage of performing noninvasive staging before treatment to guide therapeutic decisions. To harmonize staging by molecular imaging, a dedicated molecular imaging TNM staging system for PSMA PET/CT and PSMA PET/MRI has already been established (Prostate Cancer Molecular Imaging Standardized Evaluation). This was published in 2018, with a second revised edition being published in 2023 (9,39,40).

In the studies included in this systematic review, [^18^F]PSMA-1007 PET imaging has demonstrated high sensitivities and especially high specificities for the detection of PCa. This holds true for detection of the primary tumor, including EPE and SVI (T staging); the detection of pelvic LNs (N staging); and the detection of distant metastases (M staging). Unspecific bone uptake (UBU) will be addressed later in the discussion, but specificity for the detection of bone metastasis is not high.

In T staging, 13 of 20 reportings for sensitivity were higher than 60%, and 13 of 20 reportings for specificity were higher than 85%. [^18^F]PSMA-1007 outperformed [^68^Ga]PSMA-11 in terms of sensitivity (17,18). Compared with mpMRI in T staging, [^18^F]PSMA-1007 PET/CT resulted in higher sensitivities or specificities in some studies and lower sensitivities or specificities in other studies, showing a mixed picture. The difference in sensitivities and specificities is most likely explained by heterogeneities between studies, which will be further discussed later in this section.

In N staging, [^18^F]PSMA-1007 PET/CT demonstrated consistently high specificities (11/12 reportings > 85%). However, when compared with T staging, sensitivities were generally lower (6/12 reportings > 60%). Nevertheless, [^18^F]PSMA-1007 PET/CT outperformed MRI for N staging in all included studies in terms of sensitivity (22,24). Despite this fact, Hermsen et al. conclude that [^18^F]PSMA-1007 PET/CT cannot replace extended pelvic LN dissection for N staging of PCa because [^18^F]PSMA-1007 PET/CT underestimated the burden of pelvic metastasis (27). However, it has been shown that extended pelvic LN dissection can cause serious complications (41,42), and the benefit of extended pelvic LN dissection (staging) regarding oncologic outcomes and survival is controversial (43,44). [^18^F]PSMA-1007 PET/CT is a noninvasive method for N staging that can guide therapeutic decision-making without risking complications.

In M staging, [^18^F]PSMA-1007 PET/CT outperformed all conventional imaging modalities for the identification of distant metastases and is therefore a reliable and valid option for PCa M staging (31). Anttinen et al. reported that [^18^F]PSMA-1007 PET/CT can, however, produce a higher number of false-positive bone metastases, such as those caused by fractures and degenerative joint disease (31). The UBU of [^18^F]PSMA-1007 and consequently false-positive findings are well known in the community (45,46). Besides being caused by fractures or degenerative joint disease, UBU has also been suggested to have a connection with osteoporosis (47). In this regard, it should be mentioned that all approved PSMA PET tracers have specific training materials that offer guidance for accurate interpretation of PSMA PET results. Moreover, if UBU with [^18^F]PSMA-1007 occurs at primary staging, patients should be followed up dependent on their prostate-specific antigen level after curative intended therapy. Especially in primary staging of PCa, [^18^F]PSMA-1007 is the radiopharmaceutical of choice because of low or absent renal elimination, according to Evangelista et al. (48). However, UBU can also be experienced with other PSMA ligands because of window-level, higher spatial resolutions and possible other side pathologies such as bone fracture, osteoporosis, and benign bone involvement.

Besides its use in staging, [^18^F]PSMA-1007 PET/CT can also be used for grading of PCa, as shown in a recent study. Parameters assessed by PSMA PET/CT such as the SUV_max_ of the primary tumor, prostate total-lesion PSMA, and prostate PSMA tumor volume can correlate significantly with pretreatment prostate-specific antigen values as well as grading via Gleason score (49).

In several studies and settings, a combination of [^18^F]PSMA-1007 PET/CT and mpMRI is favored by several authors to further increase sensitivity and specificity and overcome limitations of the individual modalities (19,22,23). Two studies have shown that the combination of mpMRI and [^18^F]PSMA-1007 PET/CT can notably increase sensitivity for EPE and SVI T staging (22,23). Additionally, as [^18^F]PSMA-1007 PET/CT may overestimate tumor extent and MRI may underestimate it, the combination may improve lesion delineation (23).

Recently, a study has also shown that a combination of mpMRI and [^18^F]PSMA-1007 PET/CT is safe and feasible as a diagnostic procedure before prostatectomy without any biopsy, as 95.7% of index tumors found by mpMRI combined with PSMA PET/CT were consistent with pathology (50). This combination has several advantages, as prostate biopsies can lead to multiple complications, additional complexity for surgery, inaccurate pathologic information, and tissue inflammation due to the biopsy (50–55). Lastly, [^18^F]PSMA-1007 PET/MRI demonstrated high sensitivities and specificities in 2 studies (12,32), although widespread use of PET/MRI may be limited because of costs and lack of availability.

Within the studies included in this review, sensitivities and specificities were mostly high, but there was variability between the individual studies. This variability can be expected, as there were several heterogeneities between the studies, such as those regarding study design (retrospective vs. prospective), number and experience of readers, patient characteristics, PET/CT procedures, and performed histopathology procedures. In most studies, histopathology procedures (including alignment of PET/CT images with histopathology specimens) were not explained in detail. However, Zamboglou et al. have shown that different coregistration pathways to align PET/CT images with histopathology can yield quite different sensitivities and specificities (20). Additionally, cutoff SUV_max_ differed between the studies and was directly linked to sensitivity and specificity, as can, for example, be seen in the studies of Hoffmann et al. (18) and Luo et al. (16). To provide insights into risk of bias and quality of studies included in this review, we have included a table that depicts key study aspects that influence bias and study quality, such as study design, study setting, sample size, number and experience of readers, and masking (Supplemental Table 3). A main aspect to be considered is that many studies were retrospective and single-center—some with a low sample size and single readers that were not masked, as was, for example, the case in the studies of Giesel et al. (25), Kesch et al. (19), and Privé et al (21).

Regarding the analysis of publication bias, a limitation of this systematic review is that only a few studies were detected for the respective staging settings. An attempt was made to check for publication bias using funnel plots if 4 or more studies were available for a staging setting. No clear hint on publication bias was detected in the funnel plots, but as there were still only a few studies per plot, publication bias cannot be ruled out completely.

## CONCLUSION

In this systematic review and metaanalysis that included both prospective and retrospective studies, PET/CT imaging with [^18^F]PSMA-1007 provided high sensitivity and specificity in T, N, and M staging of PCa when compared with histopathologic evaluation, with comparable diagnostic accuracy to MRI, whereas other conventional imaging modalities such as bone scintigraphy or CT had lower accuracy than [^18^F]PSMA-1007 PET/CT. This modality therefore offers the possibility to perform accurate noninvasive primary T, N, and M staging before treatment in a single procedure to guide therapeutic decision-making.

## DISCLOSURE

Kambiz Rahbar reports lectureship or consulting honoraria from AAA/Novartis, ABX, ABX CRO, Amgen, Jansen-Cielag, SIRTEX, Bayer Healthcare, UroTrials, and Pharmtrace. Frederik Giesel is an advisor at ABX, SOFIE Biosciences, Telix Pharma, Rhine pharma, and α-Fusion and has a patent application for PSMA-1007 and FAP-ligands (FAPI-46, FAPI-74, and FAPI-34). Ken Herrmann reports grants or contracts from Novartis and Sofie Biosciences; consulting fees from Advanced Accelerator Applications, a Novartis company, Amgen, AstraZeneca, Bain Capital, Bayer, Boston Scientific, Convergent, Curium, Debiopharm, EcoR1, Fusion, GE HealthCare, Immedica, Isotopen Technologien München, Janssen, Merck, Molecular Partners, NVision, POINT Biopharma, Pfizer, Radiopharm Theranostics, Rhine Pharma, Siemens Healthineers, Sofie Biosciences, Telix, Theragnostics, and ymabs; payment or honoraria for educational events from PeerVoice; support for American Society of Clinical Oncology participation from Janssen; participation on a data safety monitoring board or advisory board for Fusion and GE HealthCare; and ownership of stocks in Sofie Biosciences, Pharma15, NVision, Convergent, Aktis Oncology, and AdvanCell, outside the submitted work. Mijin Yun is chief executive officer for Newcure M, Inc. Tadashi Watabe receives a joint research fund from α-Fusion. Ines Rudolph and Alexander Hoepping are employees of ABX. Tobias Maurer reports speaker fees from ABX, Astellas, Bayer, Sanofi-Aventis, and Phillips; consultant fees from ABX, Advanced Accelerator Applications International S.A., Ascenian, Astellas, Axiom, Blue Earth Diagnostics, GEMoAb, Novartis, ROTOP Pharma, and Telix; and research funding from ABX, Brainlab, Intuitive Surgical, and Telix. No other potential conflict of interest relevant to this article was reported.
